# Spectrum of different categories of Primary Immunodeficiency Disorders diagnosed at Children Hospital

**DOI:** 10.12669/pjms.41.2.9511

**Published:** 2025-02

**Authors:** Aisha Iftikhar, Mobeen Nazar, Adeela Chaudry, Ahmad Qaisar

**Affiliations:** 1 Aisha Iftikhar, FCPS, Department of Pediatrics, University of Child Health Sciences, Lahore, Pakistan; 2 Mobeen Nazar, FCPS, Department of Pediatrics, University of Child Health Sciences, Lahore, Pakistan; 3 Adeela Chaudry, FCPS, Department of Pediatrics, University of Child Health Sciences, Lahore, Pakistan; 4 Ahmad Qaisar, MBBS, FMH College of Medicine and Dentistry, Lahore, Pakistan

**Keywords:** B-Cell defect, Natural killer cell deficiency, Primary immunodeficiency, Severe combined immunodeficiency, Phagocytic defect, Flow cytometry

## Abstract

**Objective:**

To determine the frequency and spectrum of different categories of Primary immunodeficiency disorders (PIDD).

**Methodology:**

This was a prospective, observational analytical study, conducted in the Pediatric Medicine Department, University of Child Health Sciences (UCHS) from January 2021 to January 2023.We recruited 81 patients, initially suspected based on Jeffrey Modell Foundation(JMF) warning signs, followed by detailed evaluation. Descriptive statistics were applied.

**Results:**

Male patients exceeded female (47: 31). Median age of presentation was 17 months. Median diagnostic delay was 10.5 months. Need of I/V antibiotics was the most frequent JMF warning sign (88.5%). Consanguinity, previous hospital admissions, family history and sibling death were present in 80%, 78%, 54%, 37% of cases respectively. The most conspicuous clinical feature was persistent or recurrent thrush (51%). Patients were categorized into six main groups: B-Cell defect (29.5%), SCID (24.4%), CID (14.1%), T-Cell defect (12.8 %), Phagocytic defect (11.5%) and NK deficiency (7.7%). Main bulk of patients 37 (47.4 %) were in age-group up to one year. Most common site of infection was recurrent pneumonia (76%) and the least was septic arthritis (5.1%).

**Conclusion:**

PIDD should no longer be considered a rarity. B-Cell defect is the most common while earliest to diagnose are SCID and LAD. International health authorities should advocate EQUITABLE utilization of genetic testing across the globe.

## INTRODUCTION

Primary immunodeficiency disorders (PIDD) are a heterogeneous group of inherited diseases resulting from impaired function of one or more components of innate or acquired immunity rendering affected children susceptible to recurrent and life-threatening infections.^1^ Timely and precise diagnosis and treatment is imperative to a patient’s quality of life and survival.^2^ International Union of Immunological societies (IUIS) now terms PIDD as an inborn error of Immunity (IEI) or Immune dysregulation diseases as there is not only an increased susceptibility to infectious diseases but also to autoimmunity, auto-inflammatory diseases, allergy and malignancy.^3^

The IUIS expert committee has proposed phenotypic classification which compliments the genotypic classification. Consequently, in 2015, PIDD was re-categorized broadly into eight groups. Two were later added, establishing ten groups.^4^ A contemporary review depicts 485 distinct genetic disorders, as recently there has been substantial advancement in understanding the pathogenesis and diagnostic technologies unveiling underlying genetic causes of known PIDDs alongside identification of new phenotypes qualifying as PIDD.^5^ Conventionally inborn errors of immunity are considered a rare entity affecting one in 10,000 to one in 50,000 individuals.^6^ However, one contemporary study has reported the prevalence of PIDDs to be as high as one in 1200.^7^

Worldwide extensive research on multiple aspects of PIDDs is underway with the establishment of registries but at a national level, there is an extreme scarcity of research on PIDDs. We conducted this study with objective to determine the frequency and spectrum of different categories of Primary immunodeficiency at the University of Child Health Sciences (UCHS).

## METHOD

This was a prospective, observational analytical study, conducted in the Pediatric Medicine department, UCHS from January 2021 to January 2023. Data was collected on a pre-designed proforma with informed written consent. The study included both genders from one month to 16 years old. Total patients admitted during this period were 15035. Patients were scrutinized for PIDD based on 10 warning signs established by the Jeffrey Modell Foundation(JMF).^8^ Non-Probability Consecutive sampling was applied. Eighty-one suspected patients were subjected to HIV screening by ELISA. Three HIV-positive patients were referred to specified center and remaining 78 were included in study. Moreover, cases of acquired Hypogammaglobulinemia like malnutrition, loss of protein from urinary and GIT (Nephrotic syndrome, Protein-losing enteropathy) and secondary immunodeficiency related to drug intake like steroids or rituximab and malignancy were excluded after proper evaluation. Diagnosis of PIDD was established following criteria developed by the European Society for Immunodeficiencies (ESID).

### Ethical Approval:

It was approved by the IRB (Ref No.2021-214-CHICH; Dated: January 28, 2021).

Patients were subjected to complete history and physical examination. Variables like age of onset of symptoms, presentation and diagnostic delay were documented. Further scrutiny like H/O delayed umbilical cord separation, delayed shedding of primary teeth, previous history and number of admissions, prior surgery or biopsy, recurrent fractures, F/H of consanguinity, sibling death and similar illness in siblings or other family members, specifically maternal uncles, was conducted. Detailed vaccination history, specifically focusing on BCG and Polio drops, alongside any side-effects was recorded. Physical examination for Oral Thrush, hearing loss, Conjunctivitis, Arthritis, Skin manifestations including Seborrhea, Telangiectasia, Pyoderma, Abscess, Alopecia, Eczema, Scars of healed skin infection, Lymphadenopathy, Hepatosplenomegaly alongside complete systemic examinations like CVS, CNS, GIT, RESP System was carried out.

Investigations like CBC with peripheral smears, ESR, Reticulocyte count, ALC, ANC, were performed on all patients. Supportive investigations like Blood Culture, CRP, Coombs test, Serum Calcium, Phosphate, Alkaline Phosphatase, Urine complete culture and sensitivity, Chest X-Ray, Abdominal USG, Bone Marrow Examination, Evaluation of hair pigment and shaft abnormalities by microscopy, Alpha-feto protein, CT Chest/Imaging appropriate for site of infection were employed, when and where required. To accomplish the final diagnosis, specific tests like Immunoglobulin level, Flow cytometry to count CD3, CD4, CD8 (T Subset), CD19 (B Subsets), CD 56 (NK cells), CD 11b, CD 11c, CD18 (LAD1) and Dihydrorhodamine tests (DHR) were done tailored according to the history, physical examination and preliminary tests results.

Based on clinical features and available investigations, patients were diagnosed and categorized broadly into the following six groups considering the deficient component of immune system: T-Cell defects, B-Cell defects, Severe combined immunodeficiency, Combined immunodeficiency, Phagocytic defects and Natural Killer (NK) Cells deficiencies. Resource constraints refrained us from offering genetic analysis.

### Statistical Analysis:

Data was analyzed by using statistical software SPSS V26. Descriptive Statistics were used. Median was calculated for the quantitative variables. Qualitative variables were presented by calculating frequency and percentages.

## RESULTS

Results showed marked preponderance of male patients over female i.e. 47 (60 %): 31 (39 %). Median age of children at the time of presentation was 17 months with range of two to 156 months. Age of onset of symptoms varied from one to 24 months with median of four resulting in diagnostic delay ranging from zero to 144 months (10.5 months median). Minimum median delay amongst all six types was observed in SCID (four months) while maximum median delay was encountered in CID (72 months). Most frequently found JMF warning sign was need of IV Antibiotics to clear infections in 69 (88.5%) while least common was two or more sinus infections 9 (11.5%). Salient clinical features have been elaborated in Table-I.

**Table-I:** Important Clinical Features other than the main site of Infections.

Clinical Features	Exact number	%age
Delayed umbilical cord separation	Yes – 3	3.8 %
Delayed shedding of deciduous Teeth	Yes – 3	3.8 %
H/o recurrent fractures	Yes – 0	0 %
Past H/o Surgery	Yes – 7	9.0%
Past H/o Biopsy	Yes – 4	5.1%
Past H/o Hosp: admission	Yes – 61	78.2%
Family History	Yes – 42	53.8 %
Consanguinity	Yes – 63	80.8 %
H/o sibling Death	Yes – 29	37.2 %
Family members other than 1^st^ degree relative	Yes – 6	7.7 %
Sibling affected by similar illness	Yes – Confirm 13	16.7 %
	Suspected 29	37.2 %
BCG Given	Yes – 73	93.6 %
Complication after BCG	Yes – 2	2.6 %
Hepatomegaly	Yes – 13	16.7 %
Lymphadenopathy	Yes – 5	6.4 %
Thrush	Yes – 40	51.3 %
Clubbing	Yes – 9	11.5 %
Hearing impairment	Yes – 0	0%
Arthritis	Yes – 0	0%
Seborrhea	Yes – 6	10.3%
Pyoderma	Yes – 8	10.3 %
Eczema	Yes – 6	7.7 %
Petechiae	Yes – 1	1.3 %
Healing by scarring	Yes – 4	5.1 %
Telangiectasia	Yes – 2	2.6 %
Silvery Hair	Yes – 4	5.1 %
Occulocutaneous albinism	Yes – 3	3.8 %

**Fig.1:**
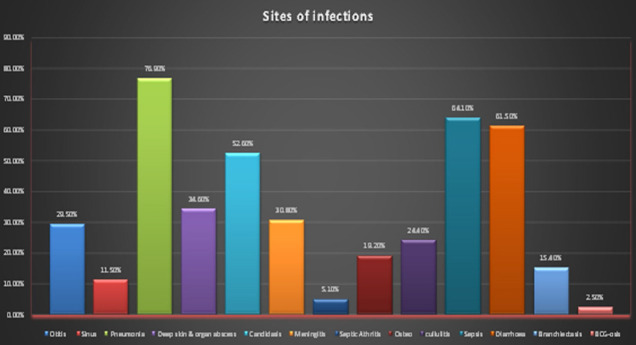
Site of infections with which patients presented.

Amid 93% cases who were given BCG vaccine, two (2.5%) developed BCG-osis and presented with complications of tuberculous lymphadenitis and disseminated tuberculosis. Both were diagnosed to have T-Cell defect later. Breakup of total patients in different age-groups as whole cohort and as main categories is demonstrated in Table-II. Categorization and different subdivisions of the main categories have been shown in Table-III. Information regarding the spectrum of sites of infections with which our patients presented is summarized in Fig.1.

**Table-II:** Distribution of patients of different age groups and whole cohort in six main categories.

Age Groups (vertically) Vs. categories breakup (Horizontally)								

	Age Group	Combined cohort (%)	Breakup of Frequency in Different Categories					
			T-Cell	B-Cell	SCID	CID	Phagocytic	NK
Breakup in age group	Up to 1yr (47.40)	37	7	9	18	1	2	0
	>1yr to 3yrs (19.2)	15	3	3	1	1	4	3
	>3yrs to 5yrs (11.5)	9	0	7	0	2	0	0
	>5yrs to 8yrs (11.5)	9	0	2	0	4	1	2
	>than 8yrs (10.3)	8	0	2	0	3	2	1
	Total (100)	78	10(12.8%)	23(29.5%)	19(24.4%)	11(14.1%)	9(11.5%)	6(7.7%)

**Table-III:** Frequency of main categories and their main subdivisions.

Disease category		Freq	%age	Male	Female
T-Cell Defect (10) (12.8%)		10	12.8%	5 (50%)	5 (50%)
B-Cell Defect (23) (29.5%)	1 IgA Deficiency	2	2.6%	2 (100%)	0
	2.Agammaglobinemia (Bruton’s dis:)	3	3.8%	3 (100%)	0
	3.Agammaglobinemia (Autosomal)	3	3.8%	0	3 (100%)
	4.Hyper IgM – Syndrome	1	1.3%	0	1 (100%)
	5.B.cell less than Normal	8	10.3%	7 (77.8%)	2 (22.2%)
	6.CVID	6	7.7%	4 (80%)	1 (20%)
SCID (19) (24.4%)		19	24.4%	9 (47.4%)	10 (52.6%)
CID (11) (14.1%)	1.Hyper IgE	7	9.0%	6 (85.7%)	1 (14.3%)
	2.Ataxia telangiectasia	2	2.6%	2 (100%)	0
	3.CID without Syndrome	2	2.6%	1 (50%)	1 (50%)
Phagocytic Defect (9) (11.5%)	1.CGD	4	5.4%	1 (25%)	3 (75%)
	2.LAD	2	2.7%	0	2 (100%)
	3.CHS	3	3.8%	2 (66.7%)	1 (33.3%)
Natural killer Cell Defect (6) (7.7%)		6	7.7%	1 (16%)	5 (83.3%)
	Total	78	100		

## DISCUSSION

Children inflicted with PIDD are susceptible to recurrent and life-threatening infections.^9^ PIDD is high in those parts of the world which harbor higher rates of consanguinity.^10^ Awareness among general public is scarce as is knowledge among physicians.^11^ Diagnosing patients of PIDD in resource-constrained countries like Pakistan bears multiple challenges at each step. Although some diagnostic facilities are available, not all exist under the roof of a single center, not even in the same city. Confounding factors like unsatisfactory sanitary conditions, rampant malnutrition and endemic infectious diseases further delays diagnosis.^12^ Accurate diagnosis demands not only an availability of lab resources but also accessible human expertise.^13^

Our results showed gender distribution (1.5:1)which is comparable to patterns observed in studies from our country^14^, as well as from other parts of the world including India and Europe.^15^ Krishna S attributes this particular phenomenon to gender discrimination in seeking medical care.^2^ In a Global systemic review, Abol Hassani has shown a comparable pattern in PIDD registries around the globe, even exceeding two times in some countries, attributed to prevalence of X-linked transmission.^16^ In the majority of these regions, this predominance holds true even when the X-linked disorders have been excluded.^2^ Our study showed that the median age of children was 17 months, the youngest diagnosed with Leukocyte adhesion defect who developed perianal abscess at one month of life and presented in 2^nd^ month.

The elapsed time between onset of symptoms and diagnosis is termed as Diagnostic Delay which is an indirect tool to determine awareness level among physicians. In the present research, median delay of 10.5 months was observed. In a Global Systemic review, diagnostic delay was compared region-wise which depicted the lowest diagnostic delay of 12 months in Iran, while maximum was in Malaysia reaching 3.78 years.^16^ Wide range of lag-time from zero to 144 months in our study not only reveals the cognizance level of physicians but also denotes that the underlying type and nature of PIDD plays a major role in identification and early referral to a tertiary care hospital.

Minimum median delay amongst all six types was observed in patients of SCID (4 months), who exhibit increasingly aggressive and distinctive features, promptly directing the physician to pursue required workup, while maximum median delay was in CID (72 months) which can be attributed to the lingering nature of its course. Qureshi S. has shown that consanguinity was present in almost all patients, corresponding with our observation.^17^ However, studies conducted around the globe show variable results; for example, Krishna S established that 47% patients had consanguineous parents.^2^ Reports from UK registry depict 2.9% patients having parental consanguinity.^18^

Our study revealed that more than half of the patients had a family history of similar illness (54%). Tipu HN has shown almost consistent findings 27(45%).^14^ While Krishna corroborates that 27% of patients had family history of PIDD.^2^ In our study, all patients were admitted with H/O recurrent infections. Similar observations have been shared by Wu Jinhong who reported 97.3% patients presented with recurrent infections. Therefore, Recurrent Infections, Consanguinity and Family History of similar illness or sibling death can be established as critical factors for suspecting and performing workup of PIDD. B-cell defect was found to be the most common PIDD type in our study. While Qureshi S has shown CGD was most common entity^17^, comparable pattern to our study has been depicted in studies from Turkey^19^ and Iran.^20^ A limitation however is that these studies also included an adult population.

In the current study, most cases within the B-cell defect group were classified as B-Cell less than normal for age. Cases of Hypogammaglobulinemia which could not be classified as CVID due to age demarcation were included in this group. Before assigning them in this category, we ruled out all other secondary causes of Hypogammaglobulinemia. These patients are currently under follow-up; repeat flow-cytometry or genetic analysis will allow us to unravel this predicament. We encountered only two patients of IgA Deficiency, contrasting European registry report which declares it to be the second most common form.^15^ This is because our data was from the in-patient department and patients with IgA deficiency usually don’t present with serious infections requiring admissions.

We encountered six patients who had NK-Cell deficiency. To conclusively declare these patients as having NK-Cell deficiency we need to repeat flow-cytometry. These patients are currently under follow-up for this purpose. In the present study, the most common infection sites encountered were lower respiratory tract infections presenting with recurrent pneumonia, followed by Diarrhea, Candidiasis, deep skin or organ abscesses in descending order of frequency. A research from Pakistan reveals that the number of diarrheal cases surpassed the cases of pneumonia followed by oral thrush and skin and deep abscess.^17^ A study done by Gupta et al. presented that lower respiratory infections and gastrointestinal illnesses are the most common infections.^21^

PIDD patients experience adverse effects following immunization (AEFI) specifically following BCGs vaccine. A report from China declares that 20.5% patients had BCG-osis.^22^ But we encountered only two patients presenting with lymphadenitis and disseminated Tuberculosis following BCG. Considering the high prevalence of immunodeficiency in our population, under-reporting is a plausible explanation of this difference. Qureshi’s study endorses similar results.^17^ BCG immunization at birth is a routine practice in Pakistan. If there is family history of PIDD or any other parameter raising suspicions of PIDD in the newborn, BCG vaccination must be deferred until immune competency is verified. Institution of newborn screening programmes may overcome this issue.

### Strengths:

At a national level there is scarcity of data regarding immunodeficiency. Our study fills this gap. To our knowledge, this is the largest collected data in two years, based on which, we have determined the spectrum and categories of PID. Furthermore, since this was conducted in a public sector hospital, diagnostic investigations like flow cytometry were provided free of cost, thus including the patients who could not afford such expensive investigations and making our sample population representative. We have included 78 patients in a two-year period and categorized them in different types with further subdivisions. Such comprehensive classification has not been done as yet in any study conducted in Pakistan.

We have documented the diagnosis of NK Cell deficiency which has not documented before as far as local and regional studies are concerned. This entity has been described in global studies. Thus its inclusion will not only expand the reader’s knowledge about this rare and elusive entity, opening new avenues for research, but also improve management for patients who might have been overlooked in the past. We have documented and compared the predilection of different types of immune deficiency in different regions of the world. This will inform physicians of the risk factors, both social and individual, that should be looked out for when performing PID workup.

### Limitations and Future Prospects:

Genetic analysis and IUIS classification could not be done. Higher evidence research on the subject would be helpful.

## CONCLUSION

PIDD should no longer be considered a rarity. B-Cell defects are the most common while earliest to diagnose are SCID and LAD. Despite increased awareness globally, diagnostic and management facilities remain scarce in Pakistan. Genetic testing carries the potential to solve many queries and can resolve the diagnostic dilemma but accessibility remains limited. International health authorities should advocate the EQUITABLE utilization of genetic testing resources across the globe to mitigate the distress and detriment experienced by patients and parents.

### Authors’ contribution:

**AI:** Conceived, designed, drafted manuscript, and conducted statistical analysis.

**MN** and **AC:** Literature Search, data collection, data entry, analysis and interpretation of data.

**AQ:** Manuscript writing, editing and revising critically for intellectual content. Revisions according to reviewer comments.

All authors have approved the final version and are responsible for accuracy and integrity of the study.
